# Author Correction: Structural basis of tRNA recognition by the m^3^C RNA methyltransferase METTL6 in complex with SerRS seryl-tRNA synthetase

**DOI:** 10.1038/s41594-025-01508-6

**Published:** 2025-02-04

**Authors:** Philipp Throll, Luciano G. Dolce, Palma Rico-Lastres, Katharina Arnold, Laura Tengo, Shibom Basu, Stefanie Kaiser, Robert Schneider, Eva Kowalinski

**Affiliations:** 1 European Molecular Biology Laboratory, Grenoble, France; 2 Institute of Functional Epigenetics, Helmholtz Zentrum Munich, Neuherberg, Germany; 3 Institute of Pharmaceutical Chemistry, Goethe University Frankfurt, Frankfurt, Germany

**Keywords:** Cryoelectron microscopy, RNA modification

Correction to: *Nature Structural & Molecular Biology* 10.1038/s41594-024-01341-3, published online 25 June 2024.

In the version of the article initially published, the sentence "We acknowledge the European Synchrotron Radiation Facility and the Deutsches Elektronen-Synchrotron for provision of synchrotron radiation facilities and we would like to thank the staff of the ESRF and EMBL Grenoble at beamline MASSIF-1, and DESY and EMBL Hamburg at beamline P13, respectively, for assistance and support” was missing from the Acknowledgements section and has now been added to the HTML and PDF versions of the article.

