# Supplementary material for: Structural basis of tRNA recognition by the m3C RNA methyltransferase METTL6 in complex with SerRS seryl-tRNA synthetase
Source: Nat Struct Mol Biol. 2024 Jun 25;31(10):1614–24. doi: 10.1038/s41594-024-01341-3 (PMC11479938; doi:10.1038/s41594-024-01341-3)
Supplement: Supplementary file 1 — Supplementary Fig. 1 and Tables 1 and 2. [file 41594_2024_1341_MOESM1_ESM.pdf]

nature structural & molecular biology

Supplementary information https://doi.org/10.1038/s41594-024-01341-3

Structural basis of tRNA recognition by the m^{3}C RNA methyltransferase METTL6 in complex with SerRS seryl-tRNA synthetase

In the format provided by the authors and unedited

Supplementary Data:

Structural basis of tRNA recognition by the m^{3}C-RNA-methyltransferase METTL6 in complex with SerRS seryl-tRNA-synthetase

Philipp Throll, Luciano G. Dolce, Palma Rico Lastres, Katharina Arnold, Laura Tengo, Shibom Basu, Stefanie Kaiser, Robert Schneider, Eva Kowalinski*

*corresponding author email: kowalinski@embl.fr

Short title: METTL6-SerRS-tRNA complex structure

Keywords: tRNA modification, cryo-EM structure, m^{3}C, methyltransferase, aminoacyl-tRNA-synthetase

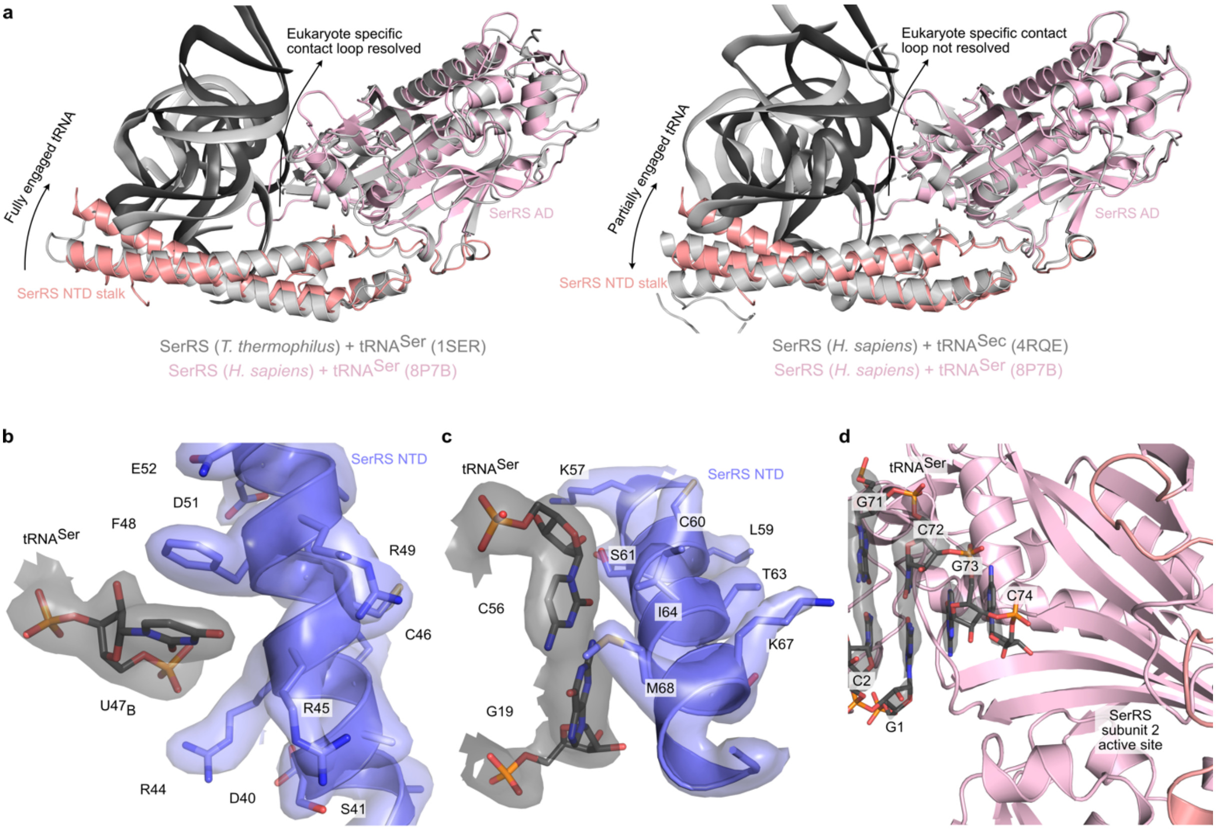

Supplementary Data Fig. 1: Binding of SerRS to tRNA^{Ser}. a Left panel: superposition of the structure of human SerRS binding to tRNA^{Ser} (this study) with the structure of *Thermus thermophilus* SerRS bound to tRNA^{Ser} (PDB-ID: 1SER, rmsd = 1.004 Å). Left panel: superposition of the structure of human SerRS binding to tRNA^{Ser} (this study) with the structure of human SerRS binding to selenocysteine tRNA (tRNA^{Sec}) (PDB-ID: 4RQE, rmsd = 0.819 Å). **b** Close-up view of the interaction between the SerRS N-terminal stalk and U47$_B$ in the variable arm of tRNA^{Ser}, with the cryo-EM map represented as a transparent surface. **c** Close-up view of the interaction between the SerRS N-terminal stalk and the G_{19}:C_{56} base pair of tRNA^{Ser}, with the cryo-EM map represented as a transparent surface. **d** Close-up view of the 3'-end of the acceptor arm of tRNA^{Ser} within the active site of SerRS, with the cryo-EM map of the tRNA acceptor arm represented as a transparent surface.

Supplementary Data Table 1: RMSD between different structures

Protein (PDB or AlphaFold DB accession)	RMSD (Å) against METTL6 bound to tRNA (8P7B)
METTL6 unbound full length (8OWX)	0.654
METTL6 unbound 40-269 (8OWY)	0.637
METTL6 unbound full length (7EZG)	0.590
METTL6 AlphaFold DB (Q8TCB7)	0.611
SpTrm141 AlphaFold DB (O74386)	0.787
SpTrm140 AlphaFold DB (Q9P7L6)	0.960
ScTrm140 AlphaFold DB (Q08641)	0.985
METTL2A AlphaFold DB (Q96IZ6)	0.974
METTL2B AlphaFold DB (Q6P1Q9)	0.992
METTL8 AlphaFold DB (B3KW44)	0.916

Supplementary Data Table 2: MS/MS parameters for quantification of modified nucleosides

Compound	Precursor Ion	Product Ion	Rt (min)	ΔRt (min)	Fragmentor (V)	Collision Energy (eV)
A	268,1	136	5,2	1	200	20
A SILIS	283	146	5,2	1	200	20
ac4C	286,1	154	5	1	85	9
ac4C SILIS	300	163	5	1	85	9
acp3U	346,1	214,1	2,3	1	95	15
Am	282,1	136	6	1	130	17
Am SILIS	298	146	6	1	130	17
C	244,1	112	2,1	1	200	20
C SILIS	256	119	2,1	1	200	20
Cm	258,1	112	4,1	1	180	9
Cm SILIS	271	119	4,1	1	180	9
G	284,1	152	4,3	1	200	20
G SILIS	299	162	4,3	1	200	20
Gm	298,1	152	5	1	100	9
Gm SILIS	314	162	5	1	100	9
I	269,1	137	4,1	1	100	10
I SILIS	283	146	4,1	1	100	10
i6A	336,3	204,1	8	1	140	17
i6A SILIS	356	219	8	1	140	17
m1A	282,1	150	2,5	2	150	25
m1A SILIS	298	161	2,5	2	150	25
m1G	298,1	166	4,9	1	105	13
m1G SILIS	314	177	4,9	1	105	13
m1I	283,1	151	4,8	1	80	12
m1I SILIS	298	161	4,8	1	80	12
m1Y	259	223	3,1	1	85	5
m22G	312,1	180	5,7	1	105	13
m22G SILIS	329	192	5,7	1	105	13
m2G	298,1	166	5,1	1	95	17
m2G SILIS	314	177	5,1	1	95	17
m3C	258,1	126	2,3	1,5	88	14
m3C SILIS	271	134	2,3	1,5	88	14
m3U	259,1	127	4,8	0,6	75	9
m5C	258,1	126	3,8	1	185	13
m5C SILIS	271	134	3,8	1	185	13
m5U	259,1	127	4,4	1	95	9
m5U SILIS	271	134	4,4	1	95	9
m66A	296	164	7,1	1	130	21
m66A SILIS	313	176	7,1	1	130	21
m6A	282,1	150	6,5	1	125	17
m6A SILIS	298	161	6,5	1	125	17
m7G	298,1	166	3,6	1	100	13
m7G SILIS	314	177	3,6	1	100	13
mcm5s2U	333,1	201	6,2	1	92	8
mcm5s2U SILIS	347,1	210	6,2	1	92	8
ncm5s2U	318,1	186	4,2	1	95	7
mcm5U	317,1	185,1	5	1	95	5
mcm5U SILIS	331	194	5	1	95	5
ncm5U	302	170	2,5	1	85	8
ncm5U SILIS	316	179	2,5	1	85	8
Q	410,2	295,1	4,3	1	115	12
t6A	413,1	281,1	5,8	1	130	9
t6A SILIS	434	297	5,8	1	130	9
U	245,1	113	3	1	95	5
U SILIS	256	119	3	1	95	5
Um	259,2	113	4,6	1	96	8
Um SILIS	271,1	119	4,6	1	96	8
Y	245,1	209	1,7	1	90	5
Y SILIS	256	220	1,7	1	90	5
ManQ	572,3	295,5	3,9	1	120	20
GalQ	572,3	295,5	4,1	1	115	20
