# Supplementary figures and images for: Structural basis of tRNA recognition by the m3C RNA methyltransferase METTL6 in complex with SerRS seryl-tRNA synthetase
Source: Nat Struct Mol Biol. 2024 Jun 25;31(10):1614–24. doi: 10.1038/s41594-024-01341-3 (PMC11479938; doi:10.1038/s41594-024-01341-3)

SDS-PAGE

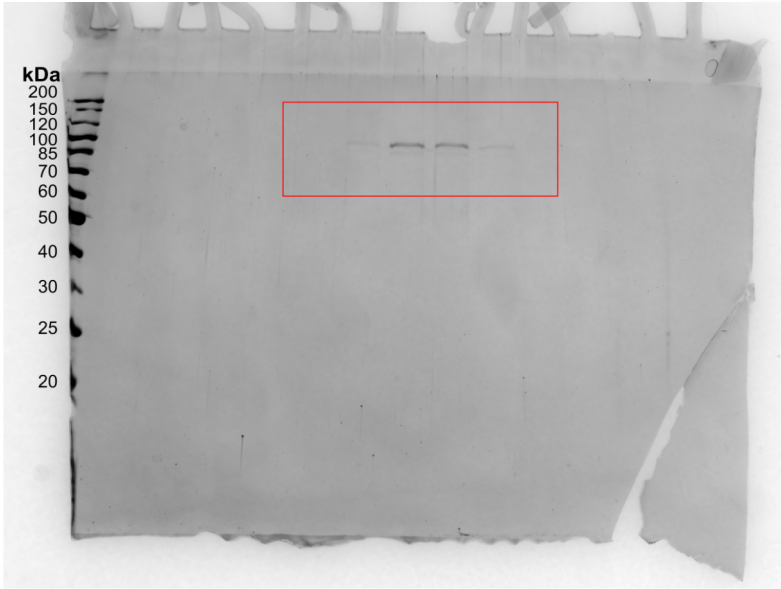

Urea PAGE

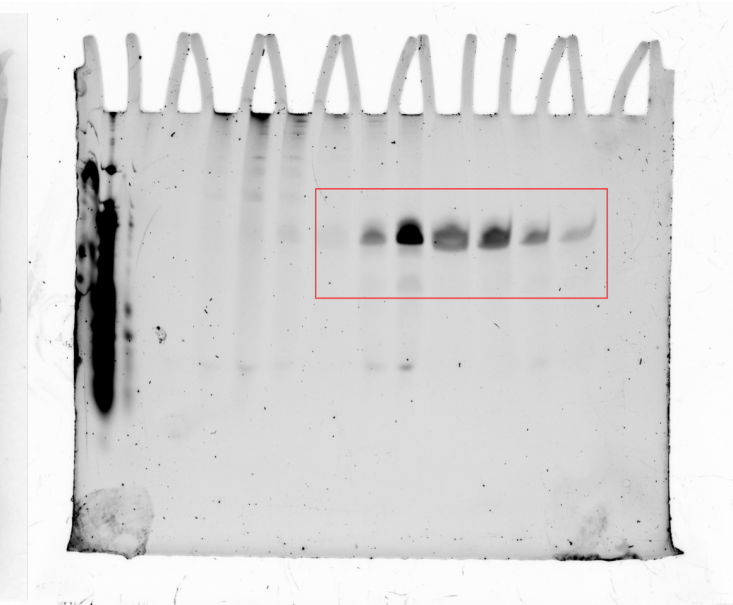

Supplement: Supplementary file 8 — Source data. [file 41594_2024_1341_MOESM8_ESM.pdf]
